# Early Experimental Report of the First 8‐Gene‐Edited Pig‐to‐Rhesus Macaque Cardiac Xenotransplantation in China

**DOI:** 10.1111/xen.70137

**Published:** 2026-05-11

**Authors:** Xianzhi Wang, Xijie Wu, Ziqiang Dai, Licheng Yan, Jie Yan, Dongsheng He, Shangxuan Li, Zhipeng Ren, Gen Zhang, Guanzheng Cui, Xin Li, Xianhua Li, Yulong Guan, Guangyu Pan, Ik Jin Yun, Wensheng Zhou, Dengke Pan, Dianyuan Li

**Affiliations:** ^1^ Department of Cardiovascular Surgery The Affiliated Suzhou Hospital of Nanjing Medical University, Suzhou Municipal Hospital Suzhou China; ^2^ Department of Cardiovascular Surgery Shengli Clinical Medical College of Fujian Medical University, Fujian Institute of Cardiovascular Disease, Fujian Provincial Hospital Fuzhou University Affiliated Provincial Hospital Fuzhou China; ^3^ Chengdu ClonOrgan Biotechnology Co, Ltd. Chengdu China; ^4^ First People's Hospital of Neijiang Neijiang China; ^5^ Extracorporeal Circulation Center National Cardiovascular Disease Center, Fuwai Hospital, Chinese Academy of Medical Sciences and Peking Union Medical College Beijing China; ^6^ Department of Cardiovascular Surgery People's Hospital of Peking University Beijing China; ^7^ Department of Surgery Konkuk University Hospital Seoul South Korea; ^8^ SIP‐UCLA Institute for Technology Advancement Suzhou China

**Keywords:** cardiac transplantation, immunosuppression, xenotransplantation

## Abstract

**Objectives:**

This study aimed to investigate the functional recovery of an 8‐gene‐edited pig heart after orthotopic xenotransplantation, the characteristics of the recipient's immune responses, and the efficacy of postoperative complication management to provide comprehensive experimental evidence for the clinical translation of xenogeneic cardiac transplantation technology.

**Methods:**

On December 27, 2025, an orthotopic heart was xenotransplanted from an 8‐gene‐edited pig to a rhesus macaque using the biatrial anastomosis technique. Postoperatively, comprehensive vital sign monitoring, immunosuppressive therapy, and complication interventions were implemented. Donor organ cardiac function, recipient immune indicators, and survival status were evaluated within 30 days after transplantation.

**Results:**

The recipient macaque survived for more than 30 days postoperatively. The cardiac function of the donor heart gradually stabilized, with the ejection fraction increasing from 58% in the early postoperative phase to 66%. No hyperacute immune rejection occurred. Postoperative complications, including infection, pleural effusion, and blood pressure fluctuations, were effectively controlled with symptomatic treatment.

**Conclusions:**

An 8‐gene‐edited pig heart demonstrates good biocompatibility in xenogeneic cardiac transplantation. Standardized surgical procedures, precise immunosuppressive regimens, and comprehensive postoperative care can effectively ensure desirable transplantation outcomes. This study offers an important technical reference for clinical xenogeneic cardiac transplantation.

## 1 Background

As a critical potential solution to the shortage of clinical cardiac donors, xenogeneic cardiac transplantation has achieved significant progress in recent years, driven by the dual advancements in gene editing technology and immunosuppressive strategies [1, 2, 3]. Pigs have become the most promising donor source for xenogeneic cardiac transplantation because of the high similarity of their cardiac anatomical structure and physiological functions to those of humans and the feasibility of reducing the immune rejection risk through genetic modification [4, 5, 6]. However, hyperacute rejection, acute vascular rejection, and chronic rejection associated with xenotransplantation remain the core obstacles limiting long‐term transplant survival rate [7, 8, 9].

With the evolution of gene editing technology, the emergence of multi‐gene‐modified pigs, such as those in which major xenogeneic antigen genes are knocked out and human complement regulatory proteins and coagulation regulatory genes are knocked in, has significantly improved the biocompatibility of the donor heart, providing key support for prolonging transplant survival time [10, 11, 12]. Among these pigs, 8‐gene‐edited pigs are engineered by knocking out major xenogeneic antigen genes, including *GGTA1*, *B4GALNT2*, and *CMAH*, while simultaneously inserting complement regulatory genes, such as *CD55* and *CD59*, coagulation function regulatory genes, such as *TBM* and *EPCR*, as well as the cytoprotective and erythropoiesis‐regulatory gene *EPO*. These modifications reduce the immune rejection risk through multiple pathways, making 8‐gene‐edited pigs a key donor model in current xenotransplantation research [13, 14, 15, 16].

In terms of surgical techniques, orthotopic cardiac transplantation can fully replace the recipient's cardiac function, making it more consistent with clinical application scenarios [17, 18, 19]. In contrast, heterotopic thoracic transplantation can maintain the recipient's native cardiac function while realizing the auxiliary support of the donor heart, which has certain advantages in perioperative hemodynamic management; however, it also involves complex vascular anastomosis and surgical positioning, and cannot completely simulate the clinical application scenario of orthotopic transplantation where the donor heart fully replaces the recipient's cardiac function [19, 20]. Therefore, optimizing the surgical workflow, immunosuppressive regimens, and perioperative management of orthotopic xenogeneic cardiac transplantation is crucial for promoting the clinical translation of this technology.

In addition, perioperative complications (e.g., infection, electrolyte imbalance, and pleural effusion) and abnormal lymphocyte proliferation during immune reconstitution also have important effects on transplantation outcomes [21, 22, 23, 24]. How to balance immunosuppression intensity and the recipient's anti‐infection ability through precise immune monitoring and individualized intervention while avoiding excessive proliferation or functional defects of immune cells is a core issue to be addressed in current pre‐clinical research [16, 25, 26]. This study used an 8‐gene‐edited pig as a donor to conduct orthotopic cardiac xenotransplantation in a rhesus macaque and systematically investigated donor cardiac function recovery, the recipient's immune response characteristics, and complication management strategies to provide more comprehensive experimental evidence for the clinical translation of xenogeneic cardiac transplantation.

## 2 Materials and Methods

### 2.1 Experimental Animals

#### 2.1.1 Donor

A male 8‐gene‐edited Bama miniature pig (*GGTA1KO*/*B4GALNT2KO*/*CMAHKO*/*hCD55*/*hCD59*/*hTBM*/*hEPCR*/*hEPO*) aged 4 months, weighing 14 kg, and with blood type O, was bred by Chengdu ClonOrgan Biotechnology Co., Ltd., using somatic cell nuclear transfer technology and housed in a designated pathogen‐free (DPF) barrier facility. Before surgery, routine blood tests and blood biochemical tests (liver and kidney function, electrolytes, etc.) were performed. Peripheral blood mononuclear cells (PBMCs) and serum were isolated and cryopreserved for subsequent use. No obvious organ dysfunction or pathogenic microbial infection was confirmed.

#### 2.1.2 Recipient

An 8‐year‐old male rhesus macaque weighing 11.6 kg and with blood type B was provided by the Laboratory Animal Research Institute of Sichuan Provincial People's Hospital. Before surgery, routine blood tests, blood biochemistry tests, and pathogen detection (TB, STLV, SRV, SIV, and BV) were conducted. Macaques with low antibody levels were screened via CDC cytotoxicity assays and IgG/IgM binding assays between monkey serum and pig PBMCs. No less than 800 mL of same‐blood‐type monkey blood was reserved for transfusion. All experiments were approved by the Institutional Animal Care and Use Committee of Sichuan Academy of Medical Sciences and Sichuan Provincial People's Hospital (Approval No. 2023009).

### 2.2 Surgical Methods

#### 2.2.1 Harvesting the Donor Heart

**FIGURE 1:**
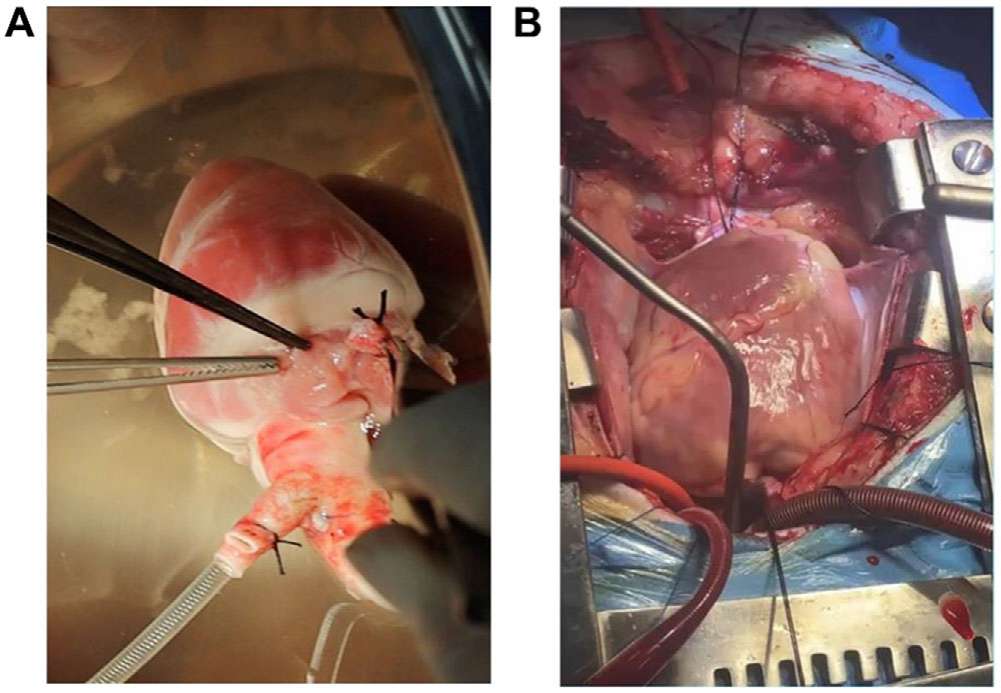
Intra‐operative status of the pig heart. A. Isolated donor pig heart. B. Donor pig heart implanted in the recipient.

The donor pig was placed in the supine position. The chest and abdominal skin were disinfected with iodophor and covered with sterile drapes. A median sternotomy was performed to enter the thoracic cavity. Bilateral costal cartilages were cut, the sternum was removed, and a retractor was used to expose the thoracic cavity and mediastinum. The pericardium was incised and suspended. After the normal cardiac structure and function were confirmed via exploration, the ascending aorta, main pulmonary artery, and superior and inferior vena cava were fully dissected and looped with tape. Systemic heparinization was performed. After the innominate artery was transected, a cold cardioplegia perfusion needle was inserted into the ascending aorta. The distal aorta was clamped between the innominate artery and the left common carotid artery, and 4°C HTK solution was rapidly perfused to induce cardiac arrest. Meanwhile, ice‐cold normal saline (4°C) was used to continuously irrigate the cardiac surface and cool it. The inferior vena cava was clamped and transected. A left atrial vent catheter was inserted through the right upper pulmonary vein for decompression. Afterward, the aorta, pulmonary artery, and superior and inferior vena cava were transected using standard procedures. The left atrial cuff containing all four pulmonary vein ostia was preserved. The heart was completely excised and immersed in 4°C sterile ice‐cold normal saline in a preservation bag for later use (Figure 1A).

#### 2.2.2 Recipient Transplantation

The bi‐atrial anastomosis technique was adopted. The recipient macaque was placed in the supine position and anesthetized with general anesthesia after routine disinfection and draping. A median sternotomy was performed layer by layer to enter the thoracic cavity, and the pericardium was suspended. Systemic heparinization was performed to establish cardiopulmonary bypass. The ascending aorta was clamped, and the cardioplegia solution was perfused. The aorta and pulmonary artery were transected above the semi‐lunar valves. The heart of the macaque was resected using the standard bi‐atrial method, preserving the posterior wall of the left atrium and part of the inter‐atrial septum tissue. The donor heart was trimmed in an ice saline basin to form left and right atrial anastomotic cuffs for convenient anastomosis. After the donor heart was placed into the recipient's pericardial cavity, anastomosis was performed sequentially: the left atrium was continuously sutured with 5–0 Prolene sutures (starting from the root of the right inferior pulmonary vein and sequentially anastomosing the bottom, lateral wall, and inter‐atrial septum). Afterward, continuous end‐to‐end anastomosis of the aorta and main pulmonary artery was completed. Finally, the right atrium was anastomosed to establish a circulatory connection. After anastomosis, a needle was inserted into the aortic root for air evacuation. The aortic cross‐clamp was released, and electric defibrillation was performed to restore cardiac contraction (Figure 1B). Gradual rewarming was performed to enter assisted circulation. After circulatory stability was achieved, cardiopulmonary bypass was terminated, and heparin was neutralized with protamine sulfate. After confirming that no active bleeding occurred, drainage tubes were placed, the sternum was closed with stainless steel wires, and the incision was sutured layer by layer. The total cardiopulmonary bypass (CPB) time for the recipient rhesus macaque was 160 min, with the total ischemic time of the 8‐gene‐edited porcine donor heart—from aortic cross‐clamping to reperfusion in the recipient—being 55 min.

### 2.3 Immunosuppressive Regimen (**Table** **1**)

#### 2.3.1 Induction Phase

A CD20 antibody (Roche [Shanghai] Co., Ltd., Shanghai, China) was intravenously infused on day −7 and day −1 before surgery. An intravenous infusion of anti‐thymocyte globulin (ATG) (Sanofi S.A., Paris, France) was administered on day −3 and day −1 before surgery, with an infusion duration of 4 h to ensure that the CD3^+^ T‐cell count was less than 500 cells/µl on the day of transplantation. An intravenous infusion of cobra venom factor (CVF) (QuidelOrtho Corporation, San Diego, CA, USA) was performed on day −7 before surgery to deplete complement. Methylprednisolone (60 mg) was intramuscularly injected daily starting on day −2 before surgery, after which the dosage was gradually reduced thereafter.

#### 2.3.2 Maintenance Phase

CD154 monoclonal antibodies were intravenously infused on day −1 before surgery, the day of surgery, and on days 3 and 7 after surgery to ensure that the drug concentration reached 500 µg/mL (detected by ELISA), followed by a once‐weekly infusion thereafter. Tacrolimus was intramuscularly injected twice daily starting on day −7 before surgery, with the blood concentration maintained at 5–10 ng/mL during the first three months and 3–8 ng/mL from month 4 to month 6. An intravenous infusion of C5 complement inhibitor was administered on days 2, 5, 10, and 21 after surgery and then once a month thereafter.

The dosage of methylprednisolone was adjusted according to the results of postoperative monitoring of immune indicators. Rituximab or rabbit anti‐human thymocyte immunoglobulin was added for enhanced immunosuppression when necessary.

**TABLE 1:** Immunosuppressive regimen for pig‐to‐rhesus macaque orthotopic cardiac xenotransplantation.

Drug agent	Time	Dosage	Route of administration
ATG	Days −3, −1	5 mg/kg	Intravenous infusion
CD20 mAb	Days −7, −1	19 mg/kg, 8 mg/kg	Intravenous infusion
CD154 mAb	Days −1, 0, 3, 7, then once weekly	25 mg/kg	Intravenous infusion
Methylprednisolone	Once daily from Day −2 onward	60 mg/day	Intramuscular injection
CVF	Day −7	100 U/kg	Intravenous infusion
Tacrolimus	Twice daily (morning and evening) from Day −7 onward	0.02–0.06 mg/kg	Intramuscular injection
C5 inhibitor	Days 2, 5, 10, 21, then once monthly	20 mg/kg	Intravenous infusion

Abbreviation: ATG, anti‐thymocyte globulin; mAb, monoclonal antibody; CVF, cobra venom factor; C5 inhibitor, complement component 5 inhibitor.

### 2.4 Postoperative Monitoring and Care

#### 2.4.1 Basic Monitoring

Vital signs (body temperature, pulse, respiration, blood pressure, oxygen saturation, and central venous pressure) were monitored daily within 30 days after surgery, and changes in the consciousness state and pupils were recorded. Cardiac function was evaluated regularly using echocardiography. Routine blood tests, blood biochemical tests, assessments of coagulation function, and measurements of myocardial markers were performed every 2–5 days. Lymphocyte subsets (expressing CD3, CD4, CD8, and CD19) were detected 1–2 times a week. Changes in the levels of CDC indicators and IgG/IgM antibodies were continuously monitored.

#### 2.4.2 Specialized Care

The patency of the left femoral triple‐lumen central venous catheter and peripheral venous indwelling needle was maintained. The infusion rate of vasoactive drugs (norepinephrine, epinephrine, dopamine, etc.) was adjusted according to the circulatory status. Mechanical ventilation was used for short‐term respiratory support after surgery, which was gradually transitioned to mask oxygen inhalation and nasal catheter oxygen inhalation. Sputum aspiration was performed regularly to maintain airway patency. Indwelling pericardial and mediastinal drainage tubes and urinary catheters were inserted. The properties and volume of drainage fluid and urine were monitored, and catheters without drainage were removed in a timely manner. Nutritional support was initiated with parenteral nutrition, including autologous blood transfusion and plasma, human albumin, and glucose injections, which was gradually transitioned to enteral nutrition with Ensure nutritional solution, apples, pears, eggs, and standard feed. Symptomatic treatments, such as closed thoracic drainage, local elevation, sedation, and analgesia, were adopted for complications such as pleural effusion, scrotal edema, and agitation.

### 2.5 Data Analysis

All the data in this study were collated and analyzed using GraphPad Prism 10.0 statistical software. Measurement data are presented according to their distribution types: normally distributed measurement data are presented as the means ± standard deviations (means ± SDs), whereas non‐normally distributed measurement data are presented as the medians (ranges). Comparison between groups of non‐normally distributed measurement data were performed using the non‐parametric Mann‒Whitney U test. Pearson's correlation analysis was used to determine the strength of the correlations between monocytes and T lymphocytes, complement C3/C4 levels, and other immune indicators. All the statistical tests were two‐tailed, with *p* < 0.05 considered to indicate a statistically significant difference and *p* < 0.01 considered to indicate a highly statistically significant difference. Repeated measurements (such as the levels of routine blood and immune indicators at different time points after surgery) were analyzed using repeated measures analysis of variance. If a time–group interaction effect existed, additional post hoc tests (with the Bonferroni correction) were conducted.

## 3 Results

### 3.1 Survival Status

**FIGURE 2:**
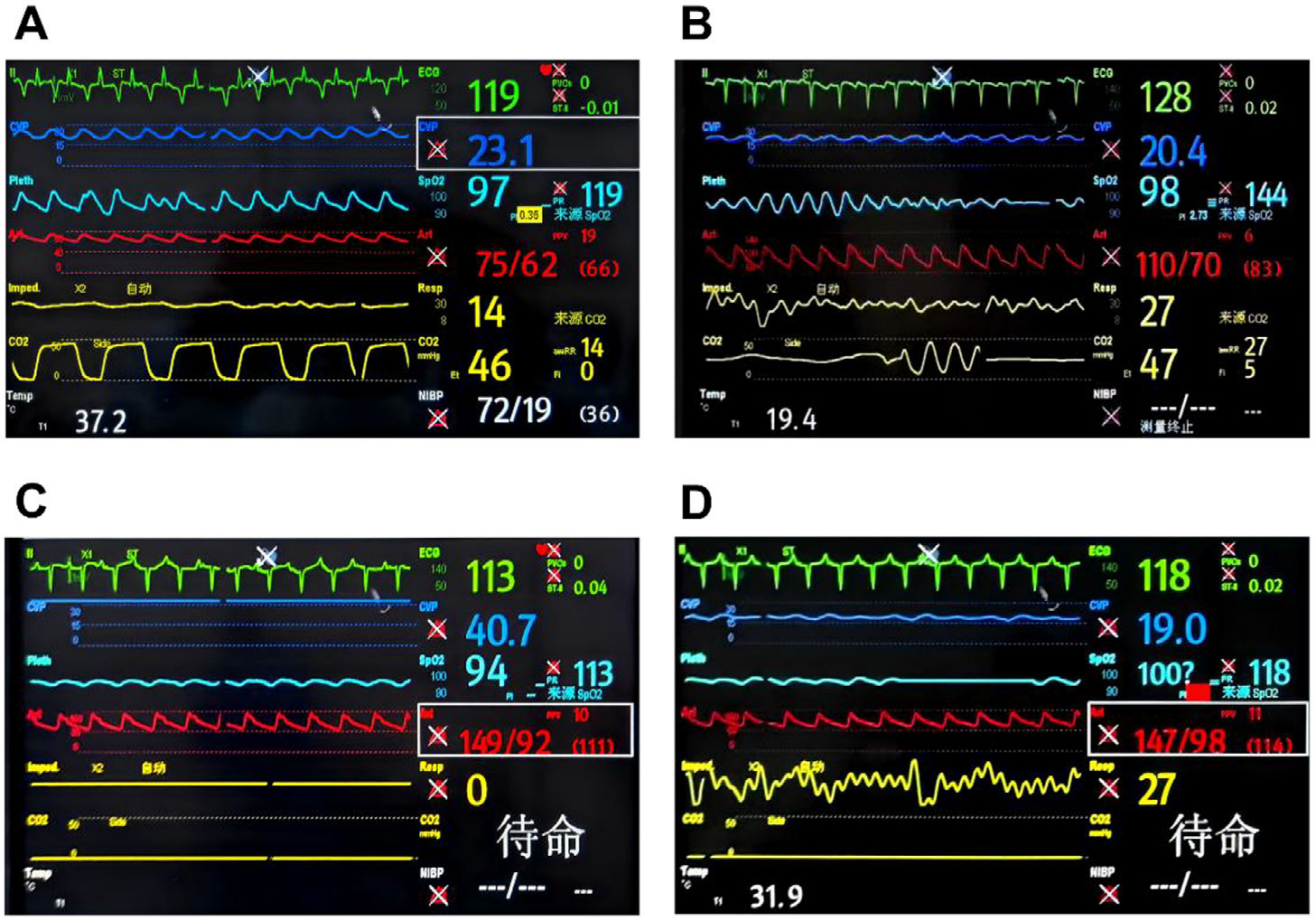
Early postoperative electrocardiographic monitoring indicators. A. Immediate postoperative electrocardiographic monitoring. B. Electrocardiographic monitoring on the first day after surgery. C. Electrocardiographic monitoring on the second day after surgery. D. Electrocardiographic monitoring on the third day after surgery.

The transplantation surgery was successful, and the donor heart resumed beating smoothly. The overall condition of the recipient macaque was good after surgery. Transient body temperature fluctuations (34.9°C–37.8°C), unstable blood pressure (75/62–147/98 mmHg), and an increased respiratory rate (up to 56 breaths/min) were observed during the early postoperative period and gradually stabilized after drug intervention and nutritional adjustment. Vital signs returned to stable levels on day 3 after surgery, and the endotracheal tube was removed (Figure 2). As of day 30 after surgery, the recipient macaque survived in good condition, with normal recovery of feeding and defecation. The recipient rhesus macaque survived for 46 days post‐transplantation and ultimately died of acute myocardial infarction (Figure S1).

### 3.2 Cardiac Function and Hemodynamics

**FIGURE 3:**
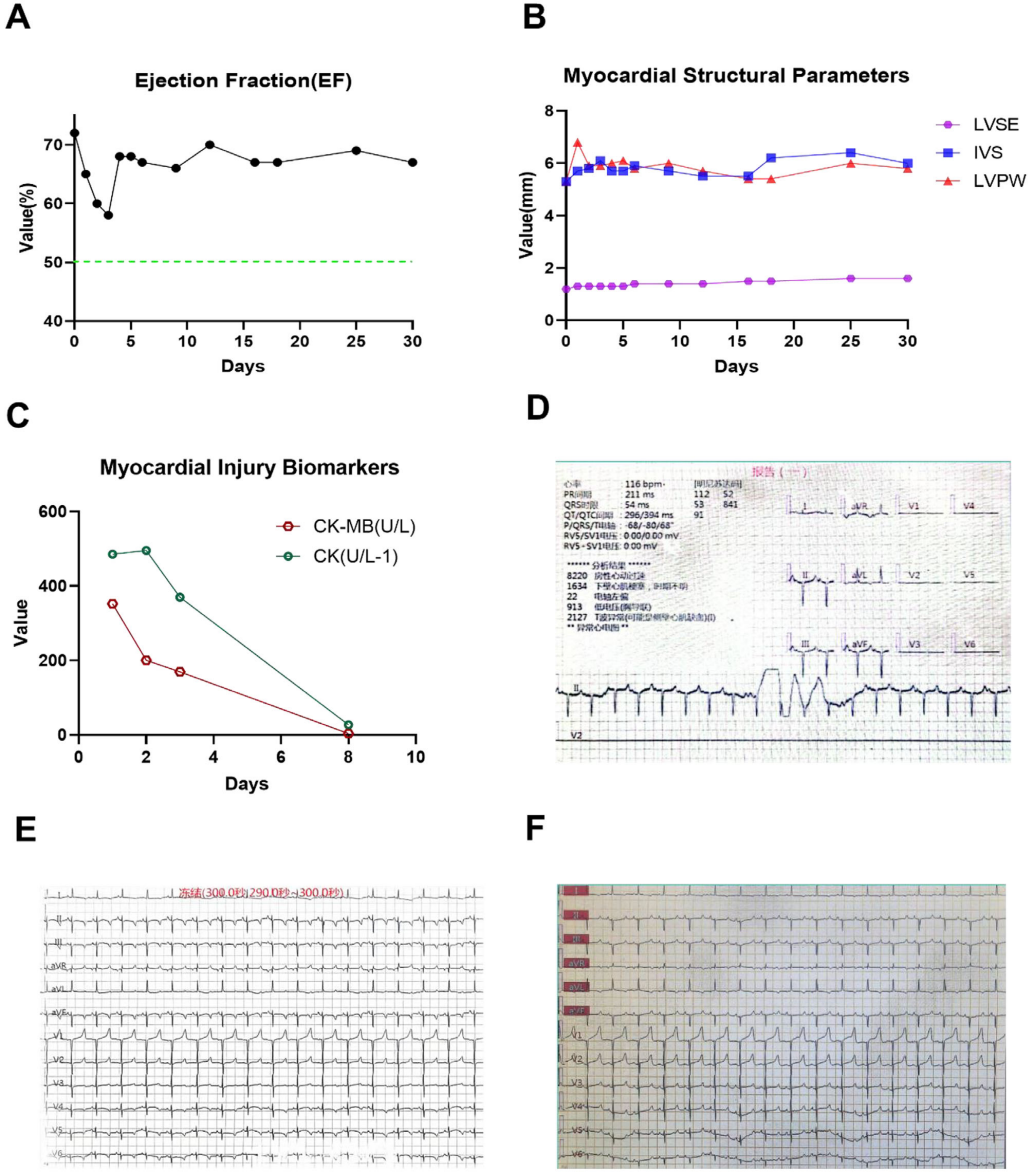
Postoperative cardiac function status. A. Stable EF values within 30 days after surgery. B. Postoperative changes in the endocardium, ventricular septum, and left ventricular posterior wall. C. Gradual decrease in the levels of myocardial injury markers postoperatively. D. Electrocardiogram recorded on the first day after surgery. E. Electrocardiogram recorded on day 17 after surgery. F. Electrocardiogram recorded on day 30 after surgery.

Postoperative echocardiographic monitoring showed that the ejection fraction (EF) of the donor heart was 58% in the early postoperative period but gradually increased to 66% by day 30 after surgery (Figure 3A). The left ventricular septal endocardial thickness was maintained at 1.3–1.4 mm, and the left ventricular posterior wall thickness was 5.8–6.8 mm, indicating a gradual improvement in overall cardiac function (Figure 3B). The cardiac function of the recipient macaque remained stable, with strong bi‐ventricular contraction and no thrombus formation. The myocardial enzyme levels (CK and CK‐MB) increased on days 1–2 after surgery, suggesting the presence of myocardial cell injury, and then gradually recovered (Figure 3C). Postoperative electrocardiograms indicated the presence of atrial tachycardia, T wave abnormalities, and left axis deviation in the early stage, and the cardiac rhythm gradually became regular after drug treatment (Figure 3D–F).

### 3.3 Laboratory Test Results

#### 3.3.1 Blood Gas Analysis

**FIGURE 4:**
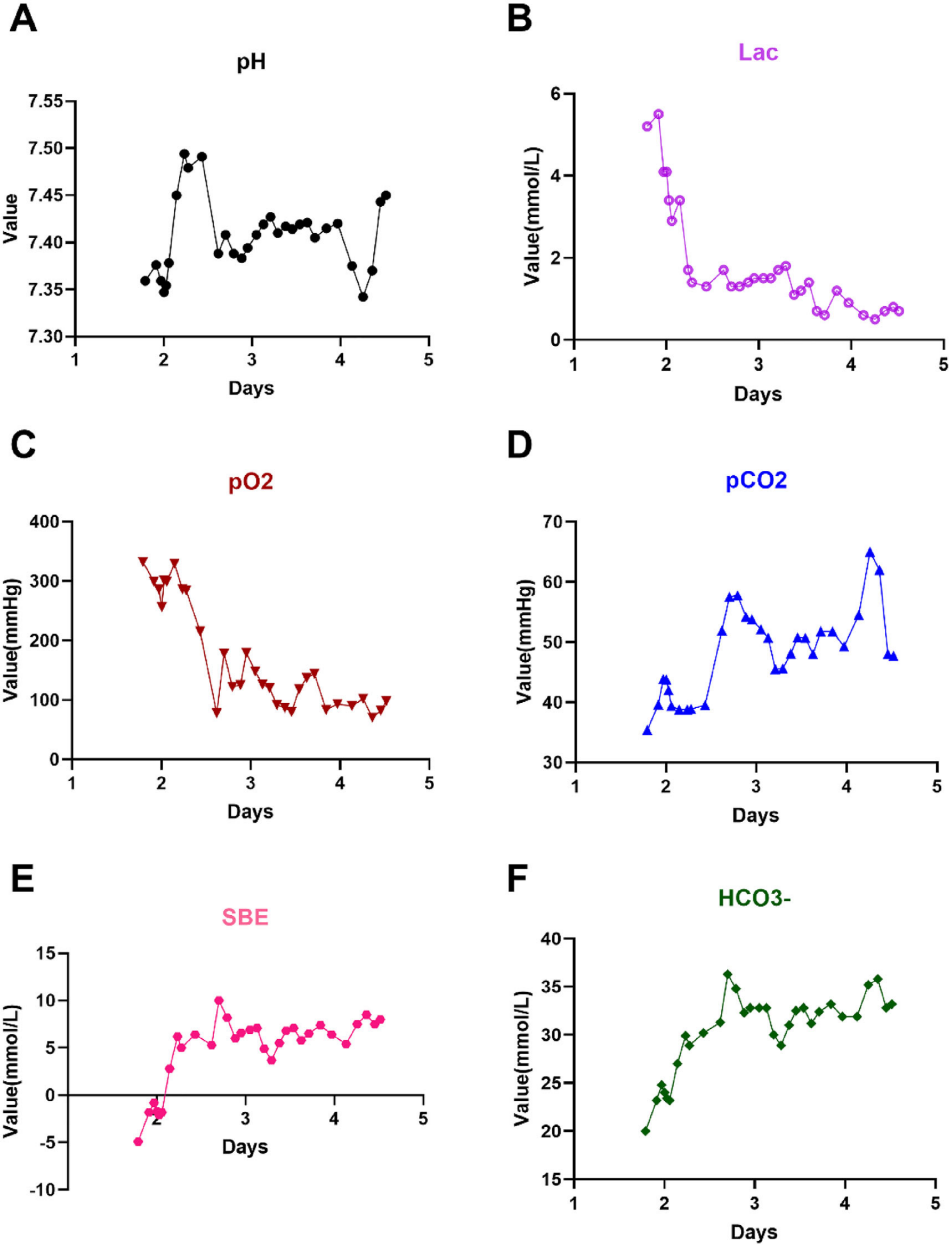
Changes in the results of the blood gas analysis within 5 days after surgery. A. Postoperative changes in the pH value. B. Postoperative changes in the lactic acid level. C. Postoperative changes in the partial pressure of oxygen; D. Postoperative changes in the partial pressure of carbon dioxide. E. Postoperative changes in standard base excess (SBE). F. Postoperative changes in the bicarbonate level.

The postoperative blood gas analysis indicated transient respiratory acidosis (peak pCO_2_: 65.0 mmHg) and hypoxemia (minimum pO_2_: 70.5 mmHg). All the indicators gradually returned to normal within 72 h after the interventions, such as adjusting the ventilator parameters, sputum aspiration, and balanced fluid infusion (Figure 4).

#### 3.3.2 Routine Blood Parameters and Electrolyte Indicators

**FIGURE 5:**
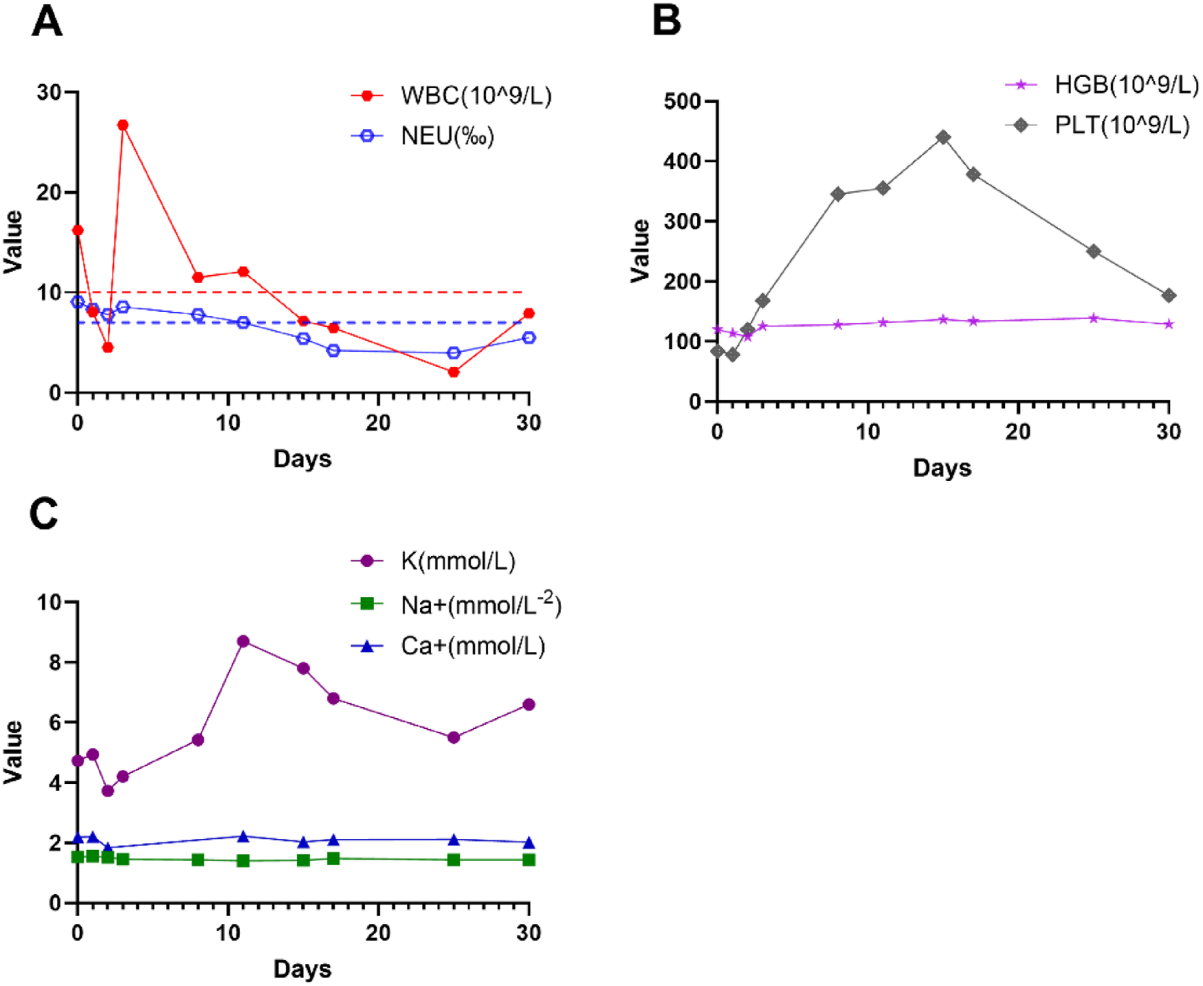
Routine blood parameters and electrolyte status. A. Postoperative changes in the white blood cell and neutrophil counts. B. Postoperative changes in the hemoglobin levels and platelet counts. C. Postoperative changes in the K^+^, Na^+^, and Ca^2^^+^ levels.

The hypersensitive C‐reactive protein concentration increased to 26.75 mg/L during the early postoperative period, indicating a postoperative inflammatory response, and then gradually decreased. The white blood cell (WBC) count was initially close to the upper limit of normal and then increased transiently. After adjusting the antibiotics (from cephalosporin to vancomycin + meropenem), the WBC count decreased rapidly and gradually returned to the normal range, and the neutrophil (NEU) count was maintained at a low level continuously (Figure 5A). The red blood cell count and hemoglobin concentration decreased slightly after surgery (hemoglobin concentration: 126 g/L), and the anemic state improved slightly by day 30 after surgery (hemoglobin concentration: 128 g/L). The platelet count was maintained within the normal range (Figure 5B). The initial potassium (K^+^) concentration ranged from 4.55.0 mmol/L, decreased transiently, then increased to a peak (approximately 8.5 mmol/L) on days 10–15, and then gradually decreased, remaining at approximately 6.5 mmol/L on day 30. No significant fluctuations in sodium (Na^+^) or calcium (Ca^2^^+^) levels were observed, indicating stable metabolism (Figure 5C).

#### 3.3.3 Immune Indicators

**FIGURE 6:**
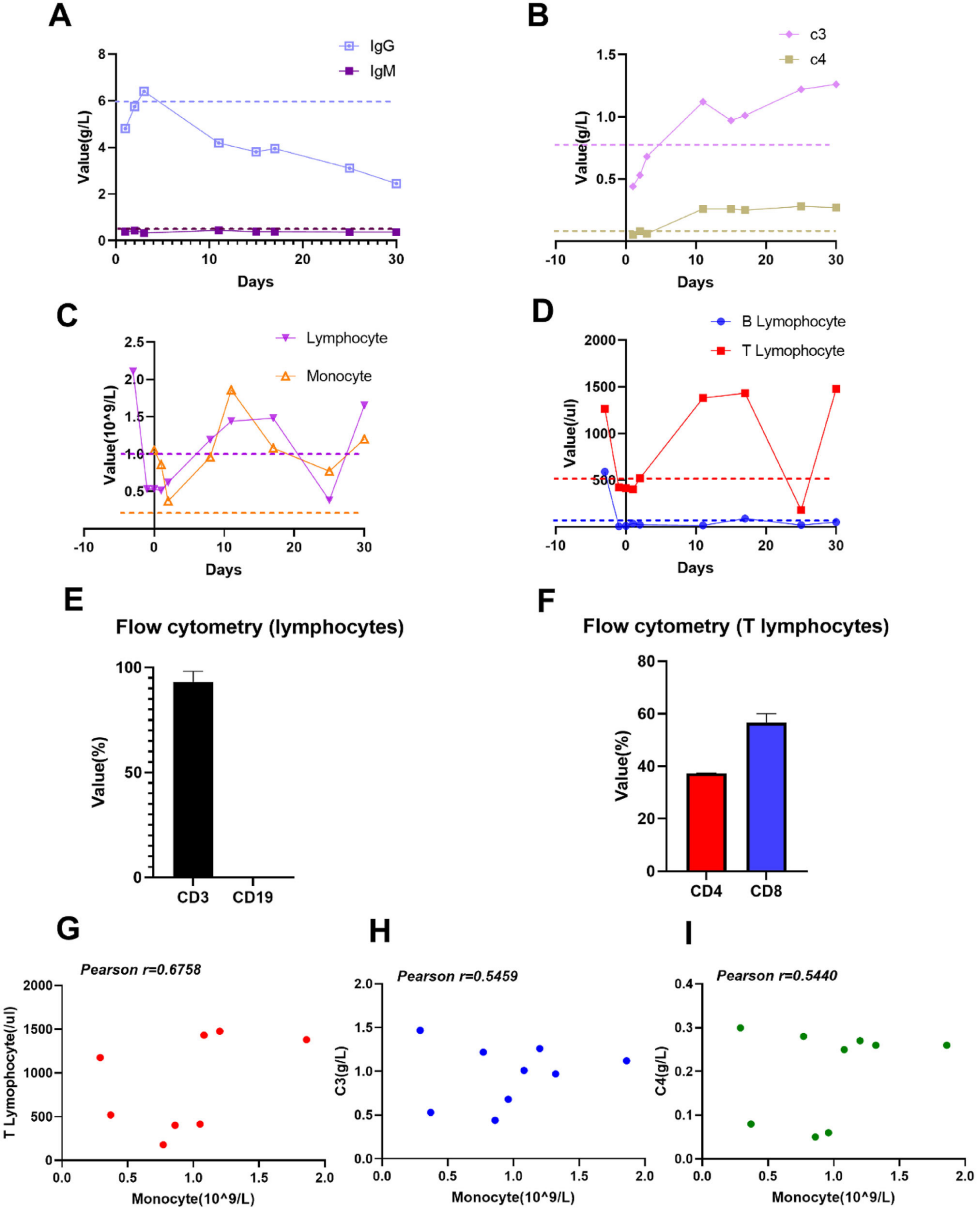
Postoperative changes in the levels of immune indicators. A. Trends for postoperative IgG and IgM levels, both of which were in a state of immunosuppression. B. Postoperative changes in complement levels. C. Postoperative changes in lymphocyte and monocyte counts. D. Postoperative changes in B and T lymphocyte counts. E. Flow cytometry analysis comparing the proportions of CD3^+^ and CD19^+^ cells. F. Flow cytometry analysis comparing the proportions of CD4^+^ and CD8^+^ T lymphocytes. G. Correlations between monocyte count and T lymphocyte counts. H. Correlations between monocyte counts and complement C3 levels. I. Correlations between monocyte counts and complement C4 levels.

The levels of immunoglobulin IgG (4.81±1.05), IgM (0.39±0.04), complement C3 (0.38 g/L), and complement C4 (0.06 g/L) were low and were related to the use of immunosuppressants (Figure 6A,B). No obvious peak in immune rejection indicators was observed, but the complement levels tended to gradually increase. In the early postoperative period, both the lymphocyte and monocyte counts were low (affected by immunosuppressants), and the lymphocyte count gradually returned to the normal range over time (the dotted line represents the reference value), with the monocyte count also increasing synchronously (Figure 6C). In the early postoperative period, both T and B cells were significantly suppressed. As time progressed, the T‐cell count gradually increased and exceeded the initial level, while the B‐cell count remained low (Figure 6D). The flow cytometry results showed that CD3^+^ lymphocytes were the main component of the increased lymphocyte population, accounting for approximately 98.5%; among these cells, CD4^+^ T cells accounted for approximately 35%, and CD8^+^ T cells accounted for approximately 60% (Figure 6E–F). The number of monocytes was significantly positively correlated with the number of T lymphocytes (*r* = 0.6758), complement C3 levels (*r* = 0.5459), and complement C4 levels (*r* = 0.5440) (Figure 6G–I).

### 3.4 Efficacy of Complication Management

#### 3.4.1 Pleural Effusion

**FIGURE 7:**
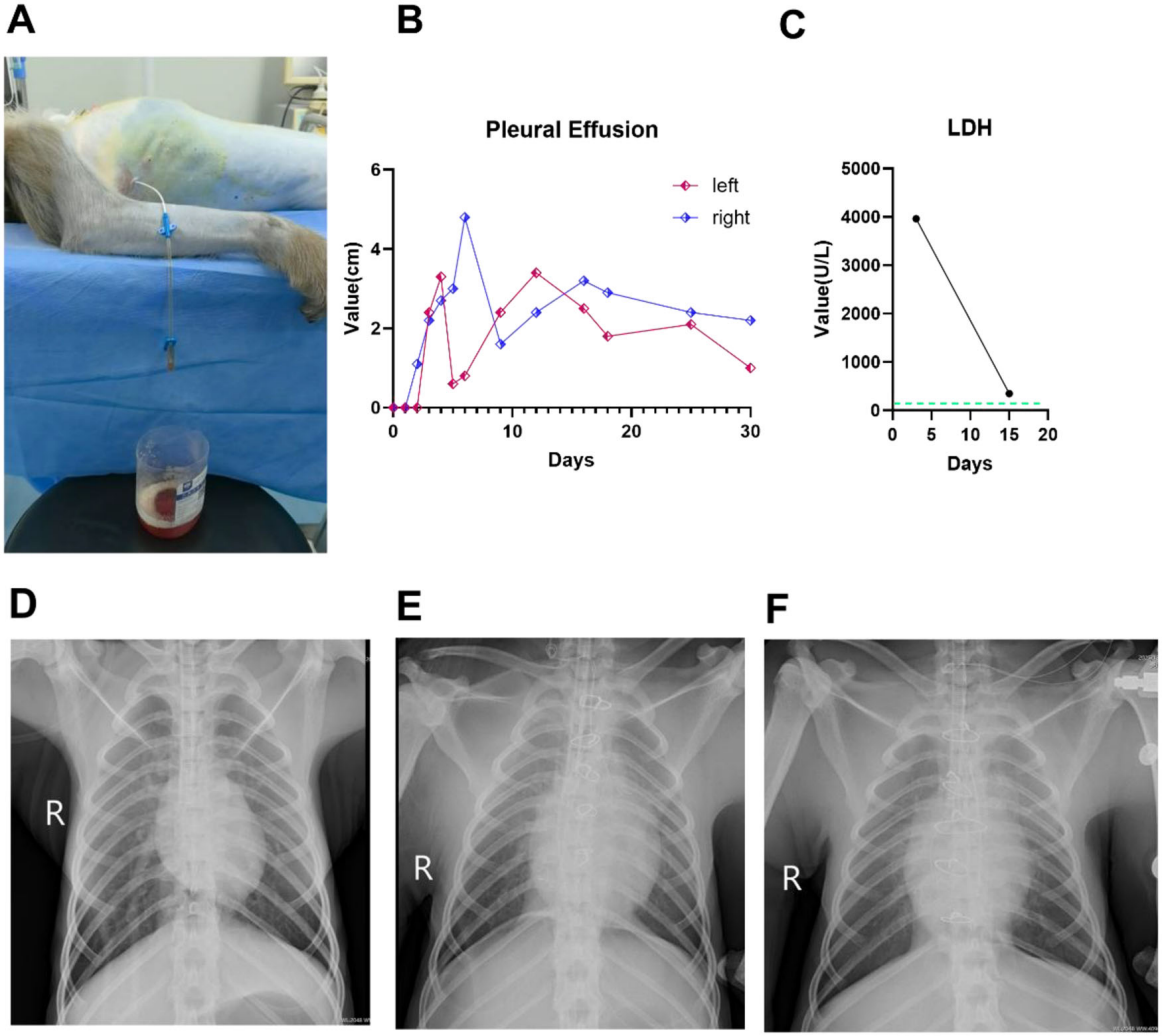
Pleural effusion and lung status. A. Bilateral pleural effusion in the recipient macaque after surgery was treated with indwelling drainage tubes for fluid drainage. B. Changes in the bilateral pleural effusion volume (gradually decreasing). C. Gradual decrease in the lactate dehydrogenase (LDH) level in postoperative pleural effusion (exudate mixed with transudate, mainly transudate). D. Preoperative chest X‐ray of the recipient macaque. E. Chest X‐ray of the recipient macaque on the first day after surgery. F. Chest X‐ray of the recipient macaque on the third day after surgery.

An ultrasound examination on day 3 after surgery revealed a small amount of bilateral pleural effusion. The effusion volume increased on December 30 (3.1/3.3 cm) (Figure 7B). After the bilateral closed thoracic drainage tubes were placed in a timely manner, the effusion gradually decreased. A chest X‐ray on day 30 after surgery showed further absorption of the pleural effusion, with clear bilateral lung fields (Figure 7D–F).

#### 3.4.2 Scrotal Edema

Obvious scrotal edema (31/25 mm) was observed on day 3 after surgery. After physical treatment with a cotton pad to elevate the scrotum, the edema gradually subsided. An ultrasound examination on day 30 after surgery showed a significant reduction in the scrotal effusion volume (5/7 mm).

#### 3.4.3 Agitation and Organ Function Injury

The recipient macaque became agitated multiple times during the first 3 days after surgery, which was effectively controlled by adjusting the infusion rate of dexmedetomidine and propofol and by administering intramuscular injection of diazepam combined with protective restraint of the limbs. Mild injuries to liver, kidney, and myocardial functions did not progress after the addition of liver‐protective drugs, myocardial nutritional drugs, and optimized circulatory management.

## 4 Discussion

The low immune rejection response and good cardiac function of the 8‐gene‐edited pig heart used as a donor were key to the success of the experiment (Figure 3). Our team used 8‐gene‐edited pigs as donors for the first time to further reduce immune rejection caused by xenotransplantation. Major xenogeneic antigen genes, such as *GGTA1*, *B4GALNT2*, and *CMAH*, were knocked out, and human complement regulatory proteins (*CD55* and *CD59*), coagulation regulatory genes (*TBM* and *EPCR*), as well as the cytoprotective/erythropoietic gene *EPO* were knocked in to further reduce the risk of immune rejection. Compared with the 5‐gene‐edited pigs (*GalT‐KO*/*β4GALNT2‐KO*/*hCD46*/*hCD55*/*hTBM*) used in previous studies by our team, the knockout of the *CMAH* gene and the knock‐in of the *CD59*, *EPO*, and *EPCR* genes were added, which optimized the biocompatibility of the donor heart more comprehensively [10, 13, 27]. *CMAH* gene knockout eliminates the expression of *Neu5Gc* in the donor heart, which is beneficial for reducing humoral immune recognition in pig‐to‐human xenotransplantation. However, in non‐human primates, such as rhesus macaques, which endogenously express *Neu5Gc* because of a functional *CMAH* gene, this modification does not necessarily reduce immunogenicity. Instead, the loss of *Neu5Gc* may lead to structural alterations or unmasking of underlying carbohydrate epitopes, which can be recognized by pre‐existing natural antibodies in the recipient [28, 29]. Previous pig‐to‐baboon xenotransplantation studies have demonstrated that *Neu5Gc* deficiency is associated with increased antibody binding and may contribute to antibody‐mediated rejection [30]. Therefore, the immune response observed in this context is more likely attributable to natural antibody recognition of altered glycan epitopes rather than a de novo adaptive immune response. This phenomenon also highlights the limitation of non‐human primate models in evaluating *Neu5Gc*‐related immunogenicity [31, 32, 33]. In our study, the recipient macaque did not present with obvious neoglycan‐induced immune rejection, which may be related to the synergistic immune suppression effect of the multi‐gene editing scheme (*CD55/CD59* knock‐in) and the perioperative immunosuppressive regimen, which neutralized the potential adverse effects of *CMAH* knockout in pig‐to‐NHP transplantation [13, 34]. CD59 is an inhibitor of the terminal complement pathway, which can prevent the formation of complement C5b‐9 (a membrane attack complex) on the donor cardiac cell membrane and avoid cell lysis and damage [11, 35]. Simultaneous knock in of *CD55* and *CD59* achieves dual inhibition of complement activation in the “early + late” stages, blocking complement‐mediated hyperacute/acute rejection responses more comprehensively than the knock in of *CD55* alone [36]. We found that the overall levels of complement C3 (0.38 g/L) and complement C4 (0.06 g/L) were low (Figure 6B). With gene knockout and drug assistance, although the complement levels increased subsequently, a significant improvement was observed compared with the complement detection results for 5‐gene‐edited pigs in previous studies, indicating that this 8‐gene‐editing scheme can improve the immune‐mediated killing effect of complement on tissues. Anemia frequently occurs in pig‐to‐NHP renal xenotransplantation due to porcine *EPO–*primate *EPO* receptor incompatibility, necessitating post‐transplant rh*EPO* administration [37, 38]. Human *EPO* knock‐in in donor pig kidneys has been shown to maintain stable hemoglobin in recipient macaques without exogenous *EPO* (*this study has not yet been published; the relevant results will be reported in our future work)*. Throughout the experiment, recipient monkey maintained stable hemoglobin levels without requiring blood transfusion, likely due to the expression of the transduced human *EPO* gene (Figure 5B). Overall, the low antigenicity of 8‐gene‐edited pigs was well verified, which increased the confidence in the results of subsequent experiments.

Donor–recipient size matching is an important consideration in cardiac xenotransplantation [39]. Unlike allotransplantation, strict size equivalence is not required; however, appropriate size matching is essential to ensure adequate hemodynamic performance and surgical feasibility [40, 41]. However, it is also important to consider that porcine donor hearts can undergo significant post‐transplant volume and weight increases [20]. Clinical reports from the first human pig heart xenotransplantation indicated that graft weight nearly doubled over time, and excessive growth contributed to complications such as left ventricular outflow tract obstruction, diastolic dysfunction, and compression of intrathoracic structures, which were important accompanying factors in graft failure in these cases [42]. Therefore, selecting an excessively large donor heart is not advisable [43]. In the present study, the donor pig (14 kg) and recipient macaque (11.6 kg) had a weight ratio of 1.21, which falls within an acceptable range and may have contributed to the stable graft function observed postoperatively.

The standardization of surgical operations is the basis for successful transplantation. We perfused the cardiac system with a 4°C HTK solution and irrigated the cardiac surface with ice‐cold normal saline for cooling to improve the ischemic tolerance of the donor heart [44]. Preserving the left atrial cuff containing all pulmonary vein ostia provided good conditions for anastomosis operations [19]. We adopted the bi‐atrial anastomosis technique to reduce the risk of stenosis after superior and inferior vena cava anastomosis and shorten the cardiopulmonary bypass time [45]. During surgery on the recipient, cardiopulmonary bypass was established accurately, and anastomosis of the atria, aorta, and pulmonary artery was completed step by step to ensure the integrity and tightness of the circulatory connection. After surgery, air evacuation from the aortic root and electric defibrillation were performed to ensure the smooth resumption of beating of the donor heart. These technical details provided a guarantee for successful transplantation.

The optimization of immunosuppressive regimens is the key to prolonging transplant survival time [11]. A combined immunosuppressive strategy of “induction + maintenance” was adopted in this study to reduce the immune rejection response [46, 47]. Before surgery, the recipient was pretreated with drugs such as an anti‐CD20 antibody, ATG, and CVF to inhibit B‐cell and T‐cell activity and the complement pathway, creating an environment of immune tolerance for transplantation [48]. After surgery, tacrolimus, anti‐CD154 mAbs, and methylprednisolone were combined with C5 complement inhibitors and other drugs to form a multitarget and comprehensive immunosuppressive network, effectively preventing hyperacute and acute immune rejection [49, 50]. Postoperative monitoring revealed that the CDC cytotoxicity percentage and IgG/IgM antibody levels were both maintained in low ranges, confirming the effectiveness of this immunosuppressive regimen. On day 17 after surgery, ATG was administered temporarily to eliminate T lymphocytes, but reactive proliferation subsequently occurred. This result is because ATG mainly rapidly clears mature T cells in peripheral blood through complement‐dependent cytotoxicity and opsonophagocytosis, but it cannot completely eliminate T‐cell precursors and memory T cells in bone marrow and lymphoid tissues [51, 52]. When the concentration of ATG gradually decreases because of metabolism in the body, residual T cells begin to proliferate and be released into peripheral blood, leading to a rebound increase in the T‐cell count [52, 53] (Figure 6C,D). Combined with the results of multiple postoperative flow cytometry, the proliferating cells were dominated mainly by CD4^+^ and CD8^+^ T lymphocytes, and B lymphocytes were in a well‐suppressed state (Figure 6E,F). Therefore, we believe that in subsequent experiments, strengthening the basic immunosuppression of T lymphocytes (tacrolimus/ciclosporin/CD154) is necessary. Notably, no inhibitor targeting monocytes was administered during the entire experimental period; thus, the monocyte count was not significantly suppressed (Figure 6C). However, monocytes interact with T lymphocytes and complement to exert their effects [35, 54, 55]. Postoperative detection and analysis showed that monocyte counts were significantly positively correlated with T lymphocyte counts, C3 levels, and C4 levels (*r* > 0.5) (Figure 6G–I). We believe that in subsequent experiments, strengthening the inhibitory effect of monocytes on overall immune rejection is necessary. At present, relevant research teams worldwide have conducted studies on reducing immune damage mediated by macrophages by knocking in human genes (including but not limited to *hCD47‐tg* and *Hcd200‐tg*) [56, 57]. Moreover, knocking out potential targets that promote the polarization of monocytes and macrophages to M1 macrophages may become a new anti‐immune rejection strategy for xenotransplantation [58, 59]. In summary, the normal cardiac function of the recipient after surgery and the ideal control of immune indicators indicated that this immunosuppressive regimen achieved good results.

Comprehensive postoperative monitoring and care are important for ensuring the survival of the recipient. The first 3 days after surgery constitute the critical period for cardiopulmonary bypass surgery [60]. In this study, a multidimensional monitoring system covering vital signs, cardiac function, laboratory indicators, and imaging examinations was established to detect problems such as blood pressure fluctuations, pleural effusion, and electrolyte imbalance in a timely manner and implement rapid interventions. For example, when the postoperative levels of infection indicators increased transiently, the antibiotics were immediately changed from cephalosporin to vancomycin + meropenem [61, 62], and the levels of the infection indicators decreased significantly after adjustment (Figure 5A). After postoperative hyperkalemia (*K* > 8.5) occurred, diuretic and glucose–insulin therapy were immediately administered [63], and the cardiac function of the recipient macaque gradually stabilized (Figure 5C). By dynamically detecting the blood gas levels of the recipient macaque, continuously adjusting and optimizing the respiratory support mode, and implementing individualized sedation and analgesia measures, the physiological homeostasis of the recipient was effectively maintained; thus, the endotracheal tube was removed on the third day after surgery (Figure 4). Notably, improving the bilateral pleural effusion of the recipient after surgery was difficult for a time (left side: 1.56 ± 1.32 cm; right side: 2.17 ± 1.43 cm), which severely affected the respiratory function of the recipient and continuously affected the stability of the internal environment (Figure 7A,B). The biochemical analysis of postoperative pleural effusion samples showed an LDH level >200, and combined with postoperative chest X‐ray, we believed that the nature of the pleural effusion was exudate mixed with transudate [64] (Figure 7C–F). With the gradual improvement in infection and nutritional status, the pleural effusion of the recipient also decreased significantly. Therefore, timely postoperative detection and active intervention were key factors for the success of the experiment.

This study has several limitations. First, single‐cell sequencing and spatial transcriptome sequencing were not completed in this case, and thus the gene expression patterns in immune cells in damaged myocardial tissues were not clarified and need to be determined in subsequent experiments. Second, the follow‐up time was limited, and the observation period needs to be extended to evaluate the risk of chronic rejection and the long‐term functional status of the donor heart. Third, porcine endogenous retroviruses (PERVs) were not measured, and the etiological surveillance system needs to be improved in subsequent studies. Fourth, due to constraints on blood volume in macaques, troponin levels were not measured, limiting the precise delineation of myocardial injury. Future studies will address this issue by incorporating troponin monitoring.

In conclusion, the successful implementation of 8‐gene‐edited pig‐to‐rhesus macaque xenogeneic cardiac transplantation confirmed the good immune compatibility of 8‐gene‐edited donor heart. Standardized surgical techniques, precise immunosuppressive regimens, and comprehensive postoperative care can effectively ensure good transplantation outcomes. In the future, further optimization of gene editing strategies, improvements in immunosuppressive regimens, and strengthened prevention and control of long‐term complications are necessary to accumulate more experimental data for the clinical translation of xenogeneic cardiac transplantation.

## Author Contributions

**Xianzhi Wang**: performed experiments, participated in transplantation surgeries, analyzed data, and wrote the manuscript. **Xijie Wu**: provided experimental guidance, analyzed data, and wrote the manuscript. **Ziqiang Dai**: performed experiments, participated in transplantation surgeries, and analyzed data. **Licheng Yan**: collected data and guided manuscript revision. **Jie Yan**: managed animals, administered immune drugs, collected/analyzed blood and other data, and guided data analysis. **Dongsheng He**: performed experiments, participated in transplantation surgeries, and analyzed data. **Shangxuan Li**: Performed experiments, participated in transplantation surgeries, and analyzed data. **Zhipeng Ren**: Performed experiments, participated in transplantation surgeries, and analyzed data. **Gen Zhang**: Performed experiments, participated in transplantation surgeries, and analyzed data. **Guanzheng Cui**: Performed experiments, participated in transplantation surgeries, and analyzed data. **Xin Li**: Performed experiments and analyzed data. **Xianhua Li**: Managed experimental facilities, participated in transplantation surgeries, and analyzed data. **Yulong Guan**: Provided experimental guidance, managed intraoperative cardiopulmonary bypass, guided postoperative monitoring, and revised the manuscript. **Guangyu Pan**: Performed experiments, participated in transplantation surgeries, and analyzed data. **Ik Jin Yun**: Guided manuscript revision. **Wensheng Zhou**: Guided manuscript revision. **Dengke Pan**: Hosted pig gene editing, formulated immune protocols, guided immune monitoring, and revised the manuscript. **Dianyuan Li**: Designed the experiment, performed transplantation surgeries, and guided manuscript writing and revision.

## Funding

Noncommunicable Chronic Diseases–National Science and Technology Major Project (2025ZD0552100); National Key R&D Program of China (2024YFC3406800); Suzhou Gusu Health Talent Program (GSWS2022065); and Suzhou “Science and Education Strengthening Medicine” Project (QNXM2024033).

## Conflicts of Interest

The authors declare that there are no conflicts of interest.

## Supporting information




**Supporting Figure S1**: Gross anatomical examination of the porcine donor heart following the death of the recipient macaque (46 days post‐transplantation) confirmed that the cause of death was acute myocardial infarction of the left ventricular anterior wall.
