# Supplementary material for: Early Experimental Report of the First 8‐Gene‐Edited Pig‐to‐Rhesus Macaque Cardiac Xenotransplantation in China
Source: Xenotransplantation. 2026 May 11;33:e70137. doi: 10.1111/xen.70137 (PMC13161745; doi:10.1111/xen.70137)
Supplement: Supplementary file 1 — Supporting Figure S1: Gross anatomical examination of the porcine donor heart following the death of the recipient macaque (46 days post‐transplantation) confirmed that the cause of death was acute myocardial infarction of the left ventricular anterior wall. [file XEN-33-e70137-s001.docx]

**SUPPLEMENTARY MATERIAL**


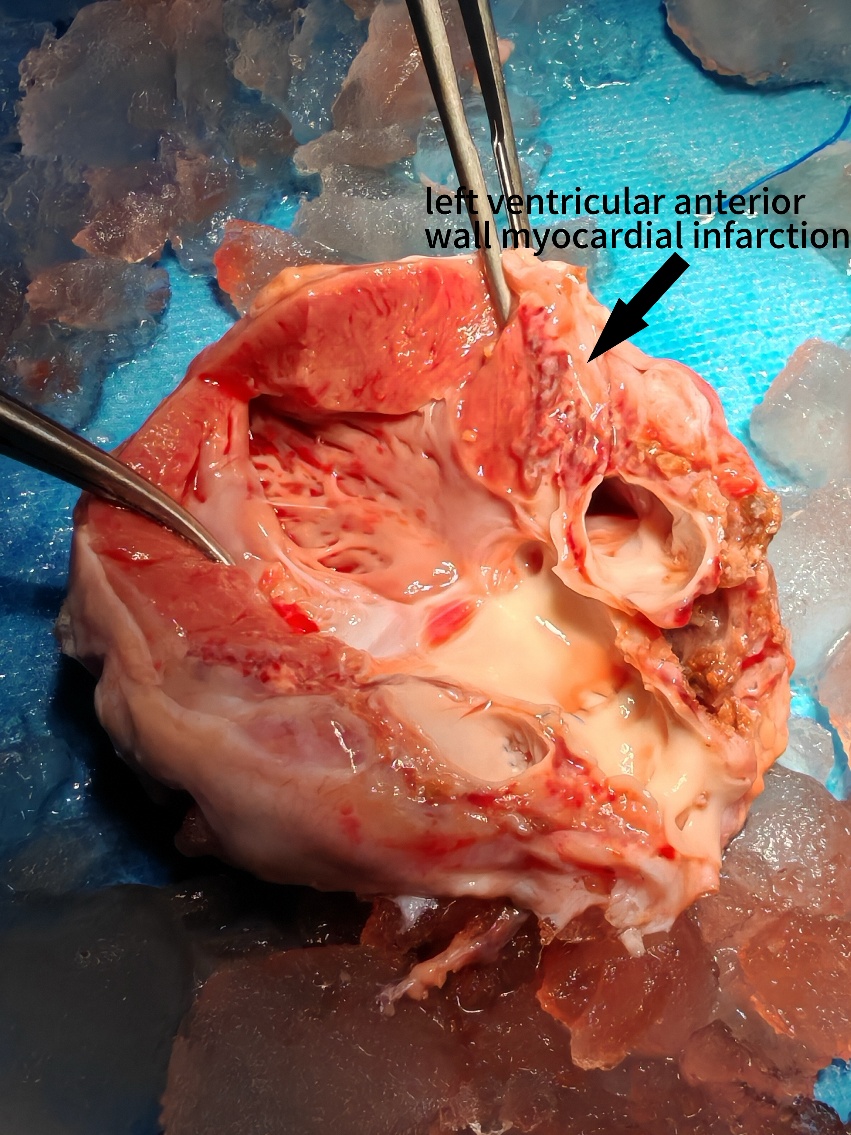


**Figure S1.** **Gross anatomical examination of the porcine donor heart following the death of the recipient macaque (46 days post-transplantation) confirmed that the cause of death was acute myocardial infarction of the left ventricular anterior wall.**
